# Supplementary material for: The impact of local assembly rules on RNA packaging in a T = 1 satellite plant virus
Source: PLoS Comput Biol. 2021 Aug 24;17(8):e1009306. doi: 10.1371/journal.pcbi.1009306 (PMC8384211; doi:10.1371/journal.pcbi.1009306)
Supplement: Attachment — Submitted filename: Reply_to_reviewers.pdf [file pcbi.1009306.s002.pdf]

Dear colleagues,

Thank you for the helpful suggestions on how to improve our submitted paper for publication. We provide below your comments (in blue) and our responses to these comments (in red).

Reviewer #1:

The authors have previously developed a stochastic model to investigate the assembly of capsid proteins around a ssRNA containing dispersed packaging signals. In this paper, they extend their previous work and consider that RNA has 30 packaging signals and investigate different pathways it connects the capsid protein subunits. The fact that viruses assemble around their own RNA rather than the host mRNA has remained a mystery despite a huge number of theoretical and experimental studies. The presence of the strong attractive interaction between packaging signals and capsid proteins in certain viruses such as MS2 and STNV has been identified as the reasons for which capsid proteins selectively package their cognate RNAs over the host mRNA.

In this paper, using stochastic modelling, the authors find that in the case of assembly of STNV, the combined effect of the packaging signal and the specific set of local rules for the RNA connecting capsid proteins significantly increases the assembly efficiency. Based on the experimental data, the authors consider that RNA containing 30 packaging signals connects 60 capsid proteins that make up the capsid. The authors find that only certain local rules are compatible with highly efficient assembly.

I think that the subject is very interesting. The authors are working on a very hot topic. The paper is relatively well-written. The findings of the authors are very interesting; thus, I recommend it for publication. However, I suggest the authors clarify several things before the publication of the paper so a broader audience can benefit from the paper.

1. The authors should explain, at least partially, which set of local rules they plan to use in the introduction of the paper. The introduction appears vague without a statement about the local rules. Maybe they can describe what the rules are about.

   We have now improved the description of the local rules and added information/data on how the local rules were chosen(Page 3 – line 82-88; Page 4/5 – line 145-152; Page 7 – line 185)

2. I understand that the authors argue that the presence of packaging signals significantly increase the efficiency of assembly of STNV. I agree with them. However, the reason that the genome and capsid proteins attract each other is electrostatic interactions. The driving force for the assembly cannot be anything but the electrostatic interaction. The packaging signal can promote the interaction and make it more efficient. I think that the authors should make it clear that the source of attraction between the genome and capsid proteins is electrostatic interaction. Maybe due to the steric interaction, a growing shell remains incomplete. Nevertheless, the reason for the aggregation of capsid proteins on the genome is electrostatics.

   We completely agree with the reviewer that the importance of electrostatic attraction is a factor in these interactions. Our model captures both an electrostatic and a sequence

specific term as a single affinity value. We have added emphasis to this fact (Page 4 – line 135-138)

3. The N-terminal domains of capsid proteins are positively charged and as such they repel each other. Thus, the electrostatic interaction between the negative charges on RNA and positive charges on N-terminals is absolutely necessary for assembly, see Phys. Rev. E 96, 022401 (2017). Do the authors consider this affinity in their model?

   As in 2, this is implicitly contained in our affinity values

4. The authors state, "in the plant viruses BMV and CCMV [9], the spontaneous encapsulation of ssRNA is driven largely by charge neutralisation interactions …" However, the viruses are almost always overcharged. The number of negative charges on RNA is always higher than the positive charges on capsid proteins, see for example Phys. Rev. E 94, 022408, (2016).

   We acknowledge that while opposite charges play a role in promoting assembly, neutralisation is not always achieved. In order to reflect that, we have made changes in the text (Page 3 – line 50-54)

5. RNA assumes different conformations inside the viral shell depending on the interaction between RNA and capsid proteins. Can the authors' model explain the different conformations of RNA observed in viral capsids, see Phys. Rev. Lett. 119, 188102 (2017).

   These pseudo-Hamiltonian paths are useful in describing where sequential contacts are made with the protein shell but do not fully describe the structure of the RNA inside. This was added to the text (Page 7 – line 212-218)

6. The relation between 3b and 3c is not very clear. I suggest that the authors expand the caption to make it clearer. It also needs to be fixed. There is no part c.

   The two subfigures display the same path on the capsid, only one does it on a 3D image of the interior of the capsid, whilst the other does it upon a 2D net of the capsid's surface. This was made clearer in the caption and the figure itself was improved (Page 11 - Fig 3 caption)

7. I think that the authors need to spend more time to explain their moves. Without knowing the previous papers of the authors, it is hard to follow the set of moves the way it is described in the paper.

   We agree with this comment and have made additions in the text (Page 3 – line 82-88; Page 4/5 – line 145-152; Page 7 – line 185)

8. The authors mention that there are many theoretical works about the non-specific interaction, but they do not refer to almost none of them. See for example Phys. Rev. E 78, 051915 (2008) and the review article, Physics Reports 847, 1-102 (2020) and many references therein.

   We have added a reference to these papers during the discussion of theoretical models (Page 2 – line 45)

9. Can the authors explain why only certain local rules are efficient? Is there a physical reason for this?

It is hard to give conclusive reasons why certain local rules are more efficient than others but it seems related to avoiding dead ends for the RNA. This can be done either by having a large number of available options at every step (e.g. 123456789) or by having a very efficient assembly strategy that the virus can use every time (e.g. 2345). The benefit of having a small number of efficient paths has been added (Page 14/15 – line 426-433)

10. Line 60 refers to Fig. 2d. I think that this is a typo. There is no Fig. 2d.

    This should have read Fig. 1d and has been changed

Reviewer #2:

The manuscript presents a very interesting study of the assembly fitness landscape for a single strand RNA satellite virus. The authors use a genetic algorithm to explore possible ways of assembling 30 helices with pairs of virus capsid proteins to assemble a complete icosahedral virus particle. The result that move {1} or {3,4} with {2,5} is necessary to generate a complete icosahedral particle is an interesting result of these simulations. The results also highlight the important of the first pentamer in forming a complete particle.

The most significant concern is that the simulations are based on reference 17, which is a non-peer-reviewed cryoelectron microscopy analysis of the STNV virus particle that has not been deposited to the Protein Data Bank. As described on page 5 lines 129-131, the cryoelectron microscopy analysis seems very interesting. However, the analysis was done in 2015 and published by the University of York, but the results are not publicly available in the Protein Data Bank. Thus, it is difficult to fully evaluate the results in the manuscript. It is also not be possible for other researchers to repeat these results or test further hypotheses based on these results.

In addition, the following points require further description and clarification prior to publication.

1. The authors could describe in more detail the result that move {2,3,4,5} is the best performing but also generates the smallest number of paths. As discussed on page 19 line 365, moves {2,3,4,5}, {1,2,3,4,5}, {2,3,4,5,8,9} all include move {2,3,4,5} and all have a strong bias toward fewer paths. This is an interesting observation, but the authors' conclusions about this result could be more clear.

   We note that also the largest possible move set {1,2,3,4,5,6,7,8,9} includes moves {2,3,4,5} but does not show the same bias during assembly as the three sets mentioned by the referee. We also note that the move sets that do not show this bias have between 7 and 9 moves compared to between 4 and 6 for those that showed bias. We therefore believe it is the number of distinct moves in the move set, rather than the inclusion of the move set {2,3,4,5} that accounts for the bias seen. This is also consistent with the fact that there is only 7% yield for move set {1,2,3,4,5}. We are not sure that the number of distinct moves is the only determinant of the outcome, but it is surely a contributing factor. We have made a corresponding comment in the text. (Page 13 – line 391-393; Page 14 – line 417-418)

2. Also on page 14 lines 392-395, the observation that there are significantly more paths that lead to complete than incomplete particles is an interesting but counter-intuitive result that

could be described in more detail. The authors could provide additional statistics to support this statement.

We observed that after the first 5-fold is completed, the number of options for the path to continue dramatically decreases based on the mutual exclusion rule. This is consistent with the fact that the only 2 complete paths that begin with a 5-fold are identical, except in how they complete the 5-fold. Unfortunately, to completely firm up the hypothesis that significantly more paths lead to complete than incomplete paths, would require us to compute a tree-like structure which would have 4^30 nodes, which is above our computational abilities. We have adjusted the text to reflect this (Page 14/15 – line 426-433)

3. The authors could provide more data demonstrating that the algorithm generates results that are truly "complete" as described on page 7 line 218. Is it truly a complete exploration of the landscape space or very efficiently sampled? The authors could provide more details about the genetic algorithm's development, including scoring criteria and benchmarking data.

   There are two distinct types of simulation in this work. The first is a complete numerical search for all combinatorically possible paths. This analysis is complete and does not rely on sampling. We then vary the probability of attachment (affinities) of the proteins to the 30 RNA binding sites, leading to a much larger configuration space. We cannot be sure that we have fully sampled this space.

   We appreciate that it was possible to only sample a relatively small part of the sequence space. However, we note that based on previous work (Viruses 17) that the numbers and positions of the high affinity PSs are most important for the outcome. Therefore, in response to the suggestion from the referee to provide statistics on the sampling, we computed the distribution of the number of high affinity signals across our sample of 1024 sequences. We established that we have a normal distribution of numbers, suggesting that we have interrogated a representative set. We also checked that the locations of the high affinity packaging signals were well sampled by computing the number of times each of the positions were occupied by a high affinity signal across the ensemble. We found that there was roughly a 50% chance (between -8kcal/M -12 kcal/M) to have a high affinity signal in each position, comared with low affinity signals (-4 to -8 kcal/M), suggesting that we sampled well from the perspective of the high affinity packaging signal positions. We have made a corresponding addition in the text (Page 8 – line 240-245)

4. On page 6 lines 178-179, the authors describe a "pseudo-Hamiltonian path." It would be helpful to explain this more and why the language of Hamiltonian paths is still helpful in this case.

   We have provided a definition of a pseudo-Hamiltonian path (Page 7 – line 194-203)

5. In the introduction, the authors refer to local assembly rules that govern virus particle assembly. It would be helpful to clearly state these rules. Do these rules have predictive value? Alternatively, is the focus more on local interactions rather than local assembly rules?

   We have better explained the nature of the local rules and the rationale for the specific rules analysed in the text. (Page 3 – line 82-88; Page 4/5 – line 145-152; Page 7 – line 185). In

answer to the reviewer's questions: the rules predict the likely assembly pathways, and therefore have predictive value. The focus of these rules is on the roles of RNA protein interactions in defining assembly pathways. We acknowledge, that protein-protein interactions and the shapes of the protein subunits also contribute to the assembly pathways. The shapes of the proteins subunits are implicitly contained in the interfaces to the other protein subunits, that impact on our move set. In that sense, local interactions play a crucial role in the local assembly rules.

6. The introduction and discussion include interesting comparisons to proposed pathways for MS2/Qβ and CCMV/BMV virus particle assembly. In the CCMV system, empty particles can be generated. Can empty particles, i.e. protein capsid only particles without genomic RNA or RNA fragments, be generated for STNV?

   Some self-assembly occurs at very high CP concentrations in the absence of RNA but this process is highly inefficient. (PhD Thesis by Rob Ford in Leeds)

Reviewer #3:

The article is well written although for non-mathematicians there are some concepts that are hard to understand. Nonetheless, this newer version of Eric and Reidun´s work includes cases in which assembly might not be mediated by packaging signals. The only improvement that I would like to see, perhaps in a second articles, is what happens when you take this model and now you also consider a distribution of the number and location of PS. What if an RNA has less PSs but there are spatially located different (closer to each other) than an RNA that has more PS but they are located far apart from each other. This question is motivated in the case of HIV-1; please see the current version of HIV-1 PS (e.g. Kevin Weeks´s, Alan Rein´s, Wei-Shau Hu´s and Micheal Summer´s work in the past 6 years). There it seems like there are multiple PS located withing a very small portion of the genome.

Anyway, this is a great article.

Thanks for the kind comments. Additional ideas for future studies/papers which we are interested in exploring using our new model.
