# Supplementary material for: The impact of local assembly rules on RNA packaging in a T = 1 satellite plant virus
Source: PLoS Comput Biol. 2021 Aug 24;17(8):e1009306. doi: 10.1371/journal.pcbi.1009306 (PMC8384211; doi:10.1371/journal.pcbi.1009306)
Supplement: Attachment — Submitted filename: response2.pdf [file pcbi.1009306.s003.pdf]

Dear Colleagues,

We again thank the reviewers for their constructive comments on our manuscript. Reviewers 1 and 3 are happy with the current version, while reviewer 2 still requests some additional changes. We address the critiques made by reviewer 2 below.

Specific comments:

Reviewer #1: The authors have addressed all the issues and comments that I had raised. To this end, I think that the paper is suitable for publication now.

RESPONSE: Thank you for your helpful feedback on the manuscript.

Reviewer #2: The revised manuscript explains points 1,2,4,5, and 6 much better. This topic is important, and the manuscript provides new insights into virus assembly. However, the authors do not address the most significant concern about basing the simulations and analysis on an unpublished cryoelectron structure showing additional density for the RNA that is not present in other deposited structures for the STNV in the Protein Data Bank. The authors do refer to PDB ID# 4v4m, but this structure does not include density for the RNA. In addition, the authors do not provide more information about the genetic algorithm, scoring criteria, or benchmarking data, as requested in point 3. The authors should consider making the algorithm and its associated datasets available on github or similar open access repository.

RESPONSE: Regarding the unpublished experimental data
We believe that there has been a slight misunderstanding regarding available experimental data on STNV and its internal RNA structure and which of these data was ultimately used by our study. The experimental data which informs our local rule model(s) have been previously published (Ford RJ, et al. Journal of Molecular Biology. 2013;425(6):1050–1064.), and the corresponding structures of the STNV particle are available for download from PDB database. The Ford structure shows RNA density underneath the two-fold axis of the capsid corresponding to 2 RNA hairpins. Our Figure 1c (with 1a and 1b) in the manuscript is based on this published data, and our mutual exclusion rule is also informed by the positional information of the two hairpins in the Ford structure. Moreover, we have used the Ford structure to calculate nearest neighbour distances between hairpins and these distances have informed our selection of potential move sets (see text on lines 92-115 of the manuscript). This is the extent of any experimental data that we have used in the paper, and this data is available as per PLOS journal requirements.

We believe our colleague has assumed that we are using unpublished experimental data in our study because we had made a brief comment regarding a new experimental structure of the STNV capsid, which at the moment is a private communication by our collaborators in Germany. This data contains additional information on genome structure in the interior of the capsid when compared with the Ford structure. This has led to the confusion that our local rules are based on the new structure as opposed to the Ford structure. Partly this is our fault, since we did not choose our words carefully. We simply wished to convey that, in future, there is potential to use our assembly model and compare the predictions of our local rule model(s) with the RNA distribution seen in the more detailed structure. The data from our German collaborators is not ours, and we have no right to publish this data nor force publication of this data. We have removed any reference to this new structure as it is not needed to determine our local rules or any other arguments in this paper.

RESPONSE: Regarding the genetic algorithm.
We believe that reviewer 2 misunderstands the nature of the genetic algorithm that we used, thinking that (1) it is more complex than it is, and (2) that there exists code which can be published. First, we note that the assembly code which performs the assembly simulations is published and available on Dykeman's research website at https://www-users.york.ac.uk/~ecd502/. To implement the genetic algorithm, we started with a random set of 1024 RNAs, each containing a different distribution of PS affinities. The implementation of the algorithm is then straight-forward. First, we submit 1024 separate computational jobs to a high-performance computing cluster, each job running a separate assembly simulation. After completion of all 1024 jobs, which takes less than a day since we are using the cluster, we sort the 1024 assembly runs from highest to lowest in terms of the total amount of successfully assembled capsid. The top 256 RNAs from the set of 1024 (in terms of assembly yield) are kept, and the remaining 768 are discarded. We generate 768 mutated sequences to replace the ones that we discarded by randomly mutating some of the packaging signal affinities in the top 256. The mutation rates are already given in the manuscript text (lines 225-251), and we refer to a previous assembly paper (Dykeman EC. Biophysical Journal. 2017;113(3):506–516) which provides the same details on these steps for those readers that want an additional example. Once the new set of 1024 (consisting of the top 256 sequences from the previous run and 768 sequences obtained by mutation of these top 256), we again submit 1024 jobs to the cluster and the process repeats. This automation process requires a specific script to run the jobs, sort the output, then resubmit the next round of jobs to the cluster. As such, this script will be cluster specific and must therefore unfortunately be written for the specific cluster architecture being used. These very widely, and therefore there is no point in providing this small wrapper script here.

However, we want to accommodate the reviewers request as much as we possibly can. We will therefore provide the initial random set of RNAs used for the genetic algorithm which we now make available for download from Dykeman's research website. Thus, we have given all feasible information for our genetic algorithm, (1) the procedural steps that we used (in the text lines-225-251), (2) the mutation rates used (line 231), and (3) the initial data set of RNAs and assembly code used to start the process (text line 249). If someone else wished to perform this procedure themselves, they could, but it would require them to code their own cluster specific script to implement the resubmission process. Implementing the script, along with this initial starting set of RNAs and assembly code, should reproduce the same results.
