# Supplementary material for: The impact of local assembly rules on RNA packaging in a T = 1 satellite plant virus
Source: PLoS Comput Biol. 2021 Aug 24;17(8):e1009306. doi: 10.1371/journal.pcbi.1009306 (PMC8384211; doi:10.1371/journal.pcbi.1009306)
Supplement: Attachment — Submitted filename: response3.pdf [file pcbi.1009306.s004.pdf]

Dear Colleagues,

We thank the reviewer for their time and comments on the manuscript. There seems to be continued confusion from Reviewer 2 about which data we have used in our computational analysis. We re-iterate again that the only data we have used is the symmetry averaged structure (PDB-id: 4BCU) that is publicly available. This has been used to locate the PS binding sites at the inner capsid surface, and from this we inferred the list of possible moves for our path analysis. From there on, we have not used any other data. In particular, we have not used any asymmetric data at all. Therefore, there really are no additional data to upload and the request from Reviewer 2: “Please make the asymmetric reconstruction and scripts publicly accessible on a site such as github or figshare.” does not make much sense to us.

We assume the reviewer might have gotten confused as we mentioned these data in an earlier version. However, since these data were private communication (at the time of James Geraets’ thesis) and it was not in our hands to make them available, we decided to omit all reference to them in the revised version of the manuscript. We made this decision, because our computational analysis and conclusions do not depend on these data. We note that we still reference James Geraets’ thesis, because he had also developed a move set (but used a completely different analysis on it), and we wanted to give him due credit for this.

We apologise to Reviewer 2 if the mention of these data in an earlier version resulted in confusion, and we hope that this explanation settles this issue. We believe that we have already taken out any reference to these data in last revision step and have therefore not made any further edits in the paper. However, as requested, we have added a reference to a GitHub repository, where the code used here is available.

With best wishes and many thanks,

Dr Eric Dykeman
