# Supplementary material for: The impact of local assembly rules on RNA packaging in a T = 1 satellite plant virus
Source: PLoS Comput Biol. 2021 Aug 24;17(8):e1009306. doi: 10.1371/journal.pcbi.1009306 (PMC8384211; doi:10.1371/journal.pcbi.1009306)
Supplement: Attachment — Submitted filename: plos2021may10.docx [file pcbi.1009306.s001.docx]

The manuscript presents a very interesting study of the assembly fitness landscape for a single strand RNA satellite virus. The authors use a genetic algorithm to explore possible ways of assembling 30 helices with pairs of virus capsid proteins to assemble a complete icosahedral virus particle. The result that move {1} or {3,4} with {2,5} is necessary to generate a complete icosahedral particle is an interesting result of these simulations. The results also highlight the important of the first pentamer in forming a complete particle.

The most significant concern is that the simulations are based on reference 17, which is a non-peer- reviewed cryoelectron microscopy analysis of the STNV virus particle that has not been deposited to the Protein Data Bank. As described on page 5 lines 129-131, the cryoelectron microscopy analysis seems very interesting. However, the analysis was done in 2015 and published by the University of York, but the results are not publicly available in the Protein Data Bank. Thus, it is difficult to fully evaluate the results in the manuscript. It is also not be possible for other researchers to repeat these results or test further hypotheses based on these results.

In addition, the following points require further description and clarification prior to publication.

1. The authors could describe in more detail the result that move {2,3,4,5} is the best performing but also generates the smallest number of paths. As discussed on page 19 line 365, moves {2,3,4,5}, {1,2,3,4,5}, {2,3,4,5,8,9} all include move {2,3,4,5} and all have a strong bias toward fewer paths. This is an interesting observation, but the authors’ conclusions about this result could be more clear.
2. Also on page 14 lines 392-395, the observation that there are significantly more paths that lead to complete than incomplete particles is an interesting but counter-intuitive result that could be described in more detail. The authors could provide additional statistics to support this statement.
3. The authors could provide more data demonstrating that the algorithm generates results that are truly “complete” as described on page 7 line 218. Is it truly a complete exploration of the landscape space or very efficiently sampled? The authors could provide more details about the genetic algorithm’s development, including scoring criteria and benchmarking data.
4. On page 6 lines 178-179, the authors describe a “pseudo-Hamiltonian path.” It would be helpful to explain this more and why the language of Hamiltonian paths is still helpful in this case.
5. In the introduction, the authors refer to local assembly rules that govern virus particle assembly. It would be helpful to clearly state these rules. Do these rules have predictive value? Alternatively, is the focus more on local interactions rather than local assembly rules?
6. The introduction and discussion include interesting comparisons to proposed pathways for MS2/Qβ and CCMV/BMV virus particle assembly. In the CCMV system, empty particles can be generated. Can empty particles, ie protein capsid only particles without genomic RNA or RNA fragments, be generated for STNV?
